# Adjunctive Hyperbaric Oxygen Therapy for a Refractory Sickle Cell‐Associated Leg Ulcer: A Case Report

**DOI:** 10.1002/ccr3.73320

**Published:** 2026-09-15

**Authors:** Jamil Wafi, Awni Alshurafa, Mohamed A. Yassin

**Affiliations:** ^1^ Department of Internal Medicine Hamad Medical Corporation Doha Qatar; ^2^ Department of Hematology Hamad Medical Corporation Doha Qatar

**Keywords:** case report, hyperbaric oxygen therapy, sickle cell disease, sickle cell leg ulcer, wound healing

## Abstract

Sickle cell leg ulcers (SCLUs) are a chronic and debilitating complication of sickle cell disease (SCD), often associated with severe pain, impaired mobility, delayed healing, and recurrence. Their management remains challenging because ulcer formation and persistence are driven by multiple overlapping mechanisms, including hemolysis‐associated vasculopathy, microvascular dysfunction, chronic inflammation, venous disease, and local tissue hypoxia. Despite standard measures such as local wound care, compression therapy, infection control, analgesia, and optimization of SCD‐directed treatment, many ulcers remain refractory, and high‐quality evidence guiding treatment is limited. We report a 44‐year‐old man with homozygous SCD (HbSS) and chronic venous disease who developed a painful refractory medial malleolar ulcer that failed to improve with conventional management. Adjunctive hyperbaric oxygen therapy (HBOT) was administered alongside standard wound care and was well tolerated, with no treatment‐related adverse events. Two months after completion of therapy, the ulcer showed near‐complete healing, accompanied by complete pain resolution and restoration of normal ambulation. This case provides additional clinical evidence that HBOT may be feasible and potentially useful in selected patients with refractory SCLUs; however, causality cannot be established from a single case, and prospective evaluation is required.

## Key Clinical Message

Refractory sickle cell‐associated leg ulcers remain challenging to treat. This case adds to the limited cumulative clinical experience with adjunctive hyperbaric oxygen therapy, which, alongside continued standard wound care and compression therapy, was followed by near‐complete ulcer healing, complete pain resolution, and restoration of normal ambulation.

## 1 Introduction

Sickle cell disease (SCD) is an inherited hemoglobinopathy characterized by chronic hemolysis, vaso‐occlusion, inflammation, and progressive end‐organ injury. Sickle cell leg ulcers (SCLUs) are among the most disabling chronic complications of SCD and may cause persistent pain, impaired mobility, prolonged healing, and frequent recurrence [1, 2].

The pathogenesis of SCLUs is multifactorial. Contributing mechanisms include endothelial dysfunction, reduced nitric oxide bioavailability, microvascular occlusion, venous insufficiency, chronic inflammation, local trauma, infection, and tissue hypoxia [3, 4]. Although local wound care, compression therapy when venous disease is present, infection control, analgesia, and optimization of SCD‐directed treatment are commonly used, high‐certainty evidence for many interventions remains limited [5, 6].

Published experience with HBOT in SCLUs remains limited to a small number of case reports. Although these reports cannot establish efficacy, the accumulation of carefully documented cases may help define patient selection, treatment protocols, safety, and clinically meaningful outcomes. We report an additional patient with HbSS, chronic venous disease, and a refractory medial malleolar ulcer who received a distinct HBOT protocol and experienced both wound improvement and functional recovery.

## 2 Case History/Examination

A 44‐year‐old man with homozygous SCD (HbSS) presented with a painful wound over the medial aspect of the right ankle that had been present for approximately one month. The pain progressively worsened and limited ambulation despite a course of oral antibiotics. He also reported generalized body and lower‐back pain consistent with prior vaso‐occlusive pain episodes.

His medical history included recurrent vaso‐occlusive crises, chronic venous insufficiency, and recurrent right lower‐limb deep vein thrombosis (DVT), with the last documented episode in 2013. He was receiving L‐glutamine and folic acid, was not taking hydroxyurea, and had not received recent transfusions. Lifelong rivaroxaban had been prescribed, although adherence was suboptimal. He smoked 10 cigarettes daily and had no history of diabetes mellitus or previous leg ulcers.

**FIGURE 1:**
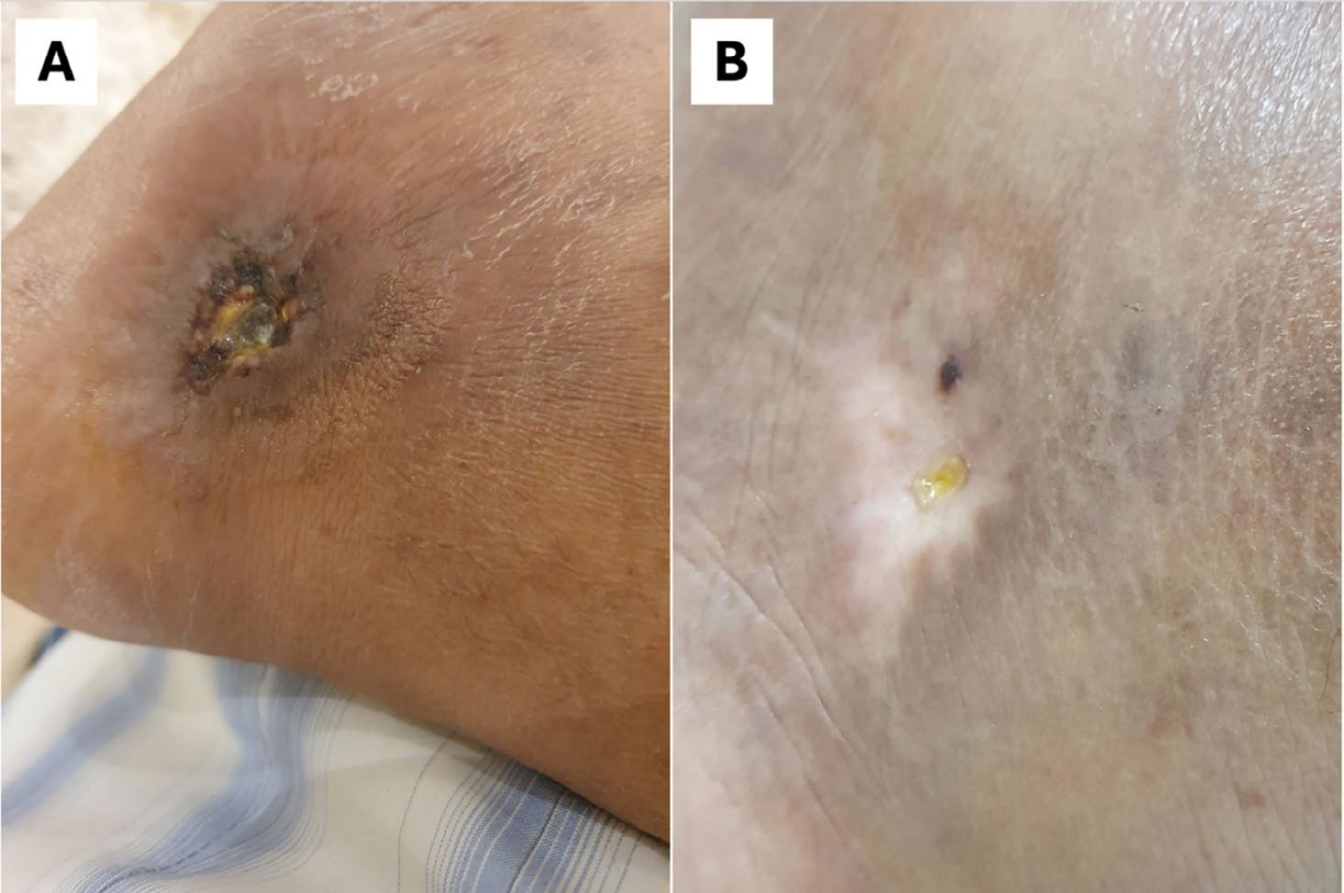
Right medial malleolar ulcer at initial presentation, showing a central necrotic eschar and surrounding tissue changes (A), and two months after completion of hyperbaric oxygen therapy, showing marked wound healing and near‐complete epithelialization (B).

On presentation, he was afebrile and hemodynamically stable. Examination revealed a 2 × 2 cm ulcer inferior to the right medial malleolus with a central necrotic eschar and surrounding fibrinous tissue (Figure 1A). The ulcer was tender and associated with mild swelling of the right foot, ankle, and lower leg. There was no purulent discharge, fluctuance, or other clear clinical evidence of active infection. Venous stasis changes and varicose veins were present, while peripheral arterial pulses were intact.

## 3 Methods

### 3.1 Differential Diagnosis

Potential alternative diagnoses included osteomyelitis, osteonecrosis, acute deep vein thrombosis, diabetes‐related ulceration, and peripheral arterial disease. These conditions were systematically evaluated and excluded through clinical assessment and diagnostic investigations.

### 3.2 Investigations

Laboratory investigations showed hemoglobin 11 g/dL, white blood cell count 15 × 10^9^/L, reticulocyte count 7%, lactate dehydrogenase 584 U/L, total bilirubin 50 micromol/L, and C‐reactive protein 5.8 mg/L. Hemoglobin electrophoresis showed HbS 79.3%, HbF 18.1%, HbA2 2.6%, and HbA 0%, consistent with HbSS. No wound culture was obtained.

Venous Doppler ultrasound of the right lower limb showed no acute DVT and only mild soft‐tissue edema. Plain ankle radiography showed no bony abnormality, and ankle magnetic resonance imaging excluded osteomyelitis and osteonecrosis.

The diagnosis of a sickle cell‐associated leg ulcer was made based on the characteristic medial malleolar location, underlying HbSS, chronic venous disease, exclusion of osteomyelitis and acute venous thrombosis, and absence of diabetes mellitus or clinically significant peripheral arterial disease.

### 3.3 Treatment

The patient was admitted and treated with oral clindamycin for possible early cellulitis, intravenous fluids, analgesia, local wound care, and compression therapy. Over five days, pain improved and ambulation was restored, allowing discharge with hematology and podiatry follow‐up.

At one‐month podiatry review, however, the ulcer persisted despite ongoing local wound care and compression. Because of persistent pain and incomplete healing, Adjunctive HBOT was recommended at hematology follow‐up.

He completed nine HBOT sessions in a multiplace chamber over three weeks, administered three times weekly. Each session lasted 120 min at a reported chamber pressure of 1.5 ATA and included two air breaks. Adjunctive HBOT was delivered with continued local wound care. Treatment was well tolerated, with no observed treatment‐related adverse events.

## 4 Conclusions and Results (Outcome and Follow‐Up)

At follow‐up two months after completing HBOT, the ulcer showed near‐complete healing with marked wound contraction and near‐complete epithelialization (Figure 1B). The patient reported complete resolution of pain, restoration of normal ambulation, and high satisfaction with the outcome. No ulcer recurrence was observed during the available follow‐up period.

The patient's clinical course from initial presentation through follow‐up is summarized in Table 1.

**TABLE 1:** Chronological timeline of the patient's clinical course, diagnostic evaluation, treatment, and outcomes.

Time point	Clinical event
Approximately 1 month before presentation	Development of a painful ulcer over the right medial ankle despite a course of oral antibiotics.
Initial presentation	Admission with worsening ankle ulcer and pain limiting ambulation. Physical examination revealed a 2 × 2 cm medial malleolar ulcer with central necrotic eschar and surrounding fibrinous tissue.
Diagnostic workup	Laboratory evaluation, venous Doppler ultrasound, ankle radiography, and MRI performed. Acute DVT, osteomyelitis, osteonecrosis, diabetes mellitus, and significant peripheral arterial disease were excluded.
Hospital admission	Treated with oral clindamycin, intravenous fluids, analgesia, local wound care, and compression therapy.
Day 5 of admission	Pain improved and ambulation restored; discharged with hematology and podiatry follow‐up.
1 month after discharge	Persistent ulcer despite ongoing wound care and compression therapy. Hyperbaric oxygen therapy (HBOT) recommended.
Following 3 weeks	Completed nine HBOT sessions (120 min each, 1.5 bar and three sessions weekly) with continued standard wound care.
2 months after HBOT completion	Near‐complete ulcer healing with marked wound contraction and epithelialization, complete pain resolution, restoration of normal ambulation, and no treatment‐related adverse events.
End of available follow‐up	No ulcer recurrence observed.

Sickle cell‐associated leg ulcers remain challenging and often require individualized multidisciplinary care. In this patient with a refractory medial malleolar ulcer, HBOT delivered with standard wound care was followed by near‐complete healing, complete pain resolution, and restoration of normal ambulation. Prospective studies are needed to clarify the efficacy, safety, optimal protocol, patient selection criteria, and durability of response for HBOT in sickle cell‐associated leg ulcers.

## 5 Discussion

This case adds to the limited cumulative clinical experience with adjunctive HBOT in refractory SCLUs. Its contribution lies not in establishing treatment efficacy, but in documenting a distinct patient with HbSS, chronic venous insufficiency, comprehensive exclusion of competing diagnoses, a different HBOT protocol, and clinically meaningful functional recovery. The ulcer occurred in a typical medial malleolar location, while acute DVT, osteomyelitis, osteonecrosis, diabetes mellitus, and clinically significant peripheral arterial disease were excluded.

SCLUs are difficult to manage because their pathobiology extends beyond a simple skin defect. Chronic hemolysis, endothelial dysfunction, impaired nitric oxide signaling, vaso‐occlusion, inflammation, venous disease, local trauma, and tissue hypoxia can interact to impair tissue repair [6]. This complexity helps explain why conventional approaches, including dressings, compression therapy, infection management, analgesia, and optimization of SCD care, may fail to achieve timely healing in some patients.

The evidence base for SCLU treatment remains limited. A Cochrane review found only six randomized trials involving 198 participants and concluded that evidence for the studied interventions was very low certainty, with no included trials addressing non‐pharmaceutical interventions such as HBOT [5]. Similarly, treatment reviews emphasize that no universally accepted, disease‐specific standard regimen exists for SCLUs and that therapy is often individualized [6].

Published experience with HBOT for SCLUs remains sparse but is gradually accumulating. Early reports described successful use of HBOT in chronic sickle cell ulcers, followed by our 2023 report and subsequent cases describing favorable outcomes with HBOT alone or in combination with skin grafting [7, 8, 9, 10]. The present case differs from our previous report because the patient had coexisting chronic venous insufficiency and a history of recurrent DVT, underwent Doppler ultrasonography and MRI to exclude important competing diagnoses, and received nine HBOT sessions over three weeks rather than four weekly sessions. The present report also explicitly documents complete pain resolution and restoration of normal ambulation. These differences broaden the cumulative clinical experience but do not establish the independent efficacy of HBOT.

The biological rationale for HBOT is plausible. By increasing dissolved oxygen in plasma, HBOT can enhance oxygen delivery to ischemic tissues even when microvascular flow and hemoglobin‐based oxygen delivery are impaired. In chronic wounds, HBOT may promote angiogenesis, fibroblast proliferation, collagen synthesis, leukocyte bactericidal activity, and edema reduction, while modulating oxidative and inflammatory signaling pathways involved in tissue repair [11, 12]. These mechanisms may be particularly relevant in SCD, where tissue hypoxia, inflammation, and microvascular dysfunction are central features of impaired healing.

The protocol in this case consisted of nine sessions over three weeks, each lasting 120 min at a reported chamber pressure of 1.5 bar with two air breaks. The patient tolerated treatment well. Published HBOT protocols for SCD‐related complications and chronic wounds vary in pressure, duration, frequency, and total number of sessions, and no standardized HBOT regimen has been established for SCLUs [11]. Ongoing investigation of HBOT in SCD, including randomized evaluation for vaso‐occlusive crises, reflects growing interest in its potential role, although these studies do not directly answer the question of efficacy in leg ulcers [13, 14].

Several limitations should be acknowledged. This is a single case report, and causality cannot be established. HBOT was administered alongside standard wound care and compression therapy; therefore, its independent contribution to healing cannot be isolated. In addition, standardized serial wound measurements were unavailable. Prospective studies and standardized treatment protocols are needed to better define the role of adjunctive HBOT in refractory sickle cell‐associated leg ulcers.

## Author Contributions

**Mohamed A. Yassin:** supervision, visualization. **Jamil Wafi:** writing – original draft, funding acquisition, conceptualization. **Awni Alshurafa:** visualization, writing – review and editing, supervision, project administration.

## Funding

No fund was received for this article. Open access publication funding was provided by Qatar National Library. The funder had no role in the study design, execution, analysis, manuscript preparation, or decision to publish.

## Ethics Statement

This case report was conducted at Hamad Medical Corporation, Doha, Qatar, in accordance with institutional policies for publication of anonymized clinical data. Formal ethics committee approval was waived for a single‐patient case report with written informed consent.

## Consent

Written informed consent was obtained from the patient for publication of the clinical details and accompanying images.

## Conflicts of Interest

The authors declare no conflicts of interest.

## Data Availability

The data that support the findings of this study are available on request from the corresponding author. The data are not publicly available due to privacy or ethical restrictions.
